# A Comprehensive Review of the Nutraceutical and Therapeutic Applications of Red Seaweeds (Rhodophyta)

**DOI:** 10.3390/life10030019

**Published:** 2020-02-26

**Authors:** João Cotas, Adriana Leandro, Diana Pacheco, Ana M. M. Gonçalves, Leonel Pereira

**Affiliations:** 1 MARE—Marine and Environmental Sciences Centre, Department of Life Sciences, Faculty of Sciences and Technology, University of Coimbra, 3001-456 Coimbra, Portugal; jcotas@gmail.com (J.C.); adrianaleandro94@hotmail.com (A.L.); dianampacheco96@gmail.com (D.P.); amgoncalves@uc.pt (A.M.M.G.); 2 Department of Biology and CESAM, University of Aveiro, 3810-193 Aveiro, Portugal

**Keywords:** rhodophyta, bioactive compounds, polysaccharides, fatty acids, pigments, phenols, applications

## Abstract

The red seaweed group (Rhodophyta) is one of the phyla of macroalgae, among the groups Phaeophyceae and Chlorophyta, brown and green seaweeds, respectively. Nowadays, all groups of macroalgae are getting the attention of the scientific community due to the bioactive substances they produce. Several macroalgae products have exceptional properties with nutraceutical, pharmacological, and biomedical interest. The main compounds studied are the fatty acids, pigments, phenols, and polysaccharides. Polysaccharides are the most exploited molecules, which are already widely used in various industries and are, presently, entering into more advanced applications from the therapeutic point of view. The focuses of this review are the red seaweeds’ compounds, its proprieties, and its uses. Moreover, this work discusses new possible applications of the compounds of the red seaweeds.

## 1. Introduction

In the last decade, there was an increasing search for new natural compounds of marine biodiversity, including microalgae, seaweeds, and invertebrates, to discover novel bioactive compounds. There is still a long way to go to discover the applications of these new natural compounds because they need to be cost efficient and economically viable from an ecological point of view. Seaweeds live in a complex and dynamic ecological location that can be denominated an extreme environment because the biotic and abiotic factors can have wide and rapid fluctuations, and the seaweeds needs to adapt rapidly. The main factors are temperature, salinity, light, pollutants, and nutrients. So, as sessile organisms, macroalgae are naturally forced to adapt to these changing environmental conditions. Then, they are able to produce a large variety of primary and secondary metabolites to respond to the surrounding environment so some of these molecules are not found in other organisms, with the specific exceptions of microalgae and some bacteria that produce identical molecules [1,2,3,4].

Due to the wide diversity of the compounds they produce, seaweeds are currently seen as promising organisms for providing new biologically active compounds for the development of novel food (nutraceutical), cosmetic (cosmeceutical), and pharmaceutical products [2,5].

Cosmeceutical compounds are substances that add a therapeutic value on cosmetics, while nutraceutical compounds are those that are added to food products and enhance their benefits. The regular consumption of nutraceutical products can be considered a dietary supplement with no harmful effects on human health. Previous researches showed that metabolites and phytochemicals extracted from seaweeds have positive effects on human health, thus decreasing symptoms of chronic diseases such as cancer, arthritis, diabetes, autoimmune, ocular, and cardiovascular diseases [6,7,8]. Although their effects are mild when compared to those of pharmaceutical products, they are considered safe, and ingestion through the daily diet as a supplement is responsible for their long-term physiological health benefits [6].

Nowadays, the interest in nutraceuticals food products is increasing due to its health beneficial properties and consumer awareness. On a research level, their potential as a treatment for chronic diseases is being investigated on a large-scale basis [9,10,11].

Seaweeds are rich in some health-promoting molecules such as ω-3 fatty acids, essential amino acids, vitamins A, B (B_1_, B_2_, B_9_,B_12_), C, D, E, K, essential minerals (calcium, iron, iodine, magnesium, phosphorus, potassium, zinc, copper, manganese, selenium, and fluoride) and also in dietary fibers [12,13,14,15].

One of the problems related to seaweed consumption is the quality of the algal biomass and the necessary quantity to prevent environmental pressures of its growing demand [16]. The seaweed global market is a sector in expansion, with an annual growth rate of 8.9%. Currently, it is evaluated on USD 11.7 billion and the previsions indicate that by 2024 it will reach the value of USD 22.13 billion [17,18]. With the FAO (Food and Agriculture Organization of the United Nations) reporting four species/genus of Rhodophyta being intensively cultivated, *Porphyra* spp., *Eucheuma* spp., *Kappaphycus alvarezii*, and *Gracilaria* spp., with nearly 18.5 thousand tonnes produced in 2016, 85% of cultivated seaweed was from China and Indonesia. These seaweeds are cultivated for the hydrocolloid and food industry [19].

**Figure 1:**
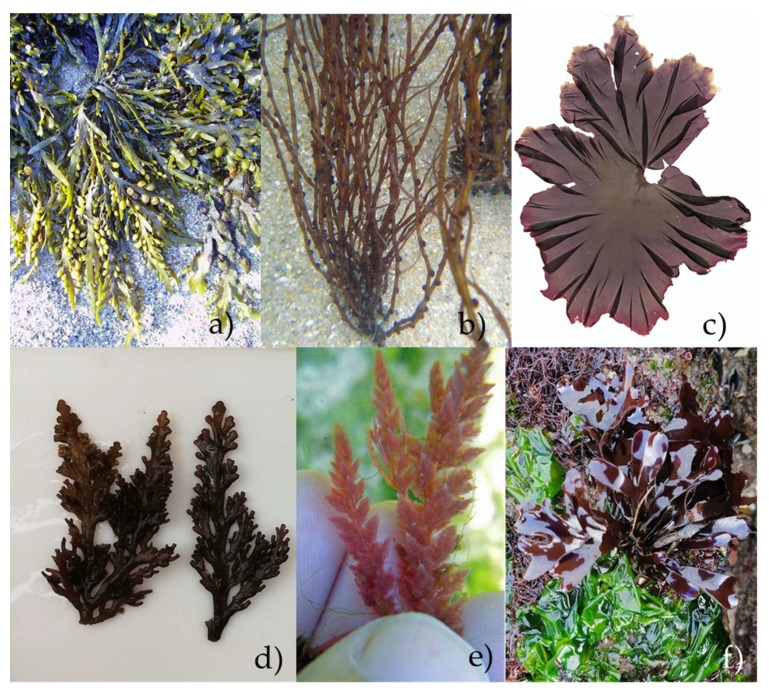
Seaweeds with potential: (**a**) *Ascophyllum nodosum* (Phaeophyceae) on rockyshore; (**b**) *Gracilaria gracilis* in Cabo Mondego beach, Figueira da Foz, Portugal; (**c**) Illustration of *Porphyra* sp.; (**d**) *Osmundea pinnatifida* specimens collected from Figueira da Foz, Portugal; (**e**) *Asparagopsis armata*, from Peniche, Portugal; (**f**) *Chondrus crispus*, in wild habitat, Cabo Mondego Beach, Portugal.

Cultivation can be an option for providing a reliable, harmless, and sustainable seaweed biomass. Thus, with the focus on the characterization of the natural variability of the compounds and culture systems’ optimization, seaweed aquaculture has been done with several species, such as *Kappaphycus alvarezii* (Rhodophyta) and *Saccharina latissima* (Phaeophyceae) [20,21]. On the other hand, there is also the possibility of exploring the potentialities of wild macroalgae, that may have rapid growth rate or have a high and consistent biomass in the wild, which happens with *Ascophyllum nodosum* (Phaeophyceae) (Figure 1a) and *Gracilaria gracilis* (Rhodophyta) (Figure 1b).

Red seaweeds (Rhodophyta) are the group of seaweeds with the potential to be one of the main groups to obtain natural compounds in the future by cultivation methods, not only in phycocolloid industry, which already happens nowadays, but in pigments and others industries, such as the substitute of animal-based gelatin with seaweed polymers, or textile industry for natural pigments [19]. Moreover, the current methods to extract and purify some of the compounds already discovered are costly and economically unviable for scale-up; however, the biopolymers (polysaccharides) produced by red seaweeds that are already applied in various industries, such as pharmaceutical, food, and biomedical industries, are an exception to this fact. Still, the research in this area is conducted to obtain more efficient and ecological extraction methods [21,22].

Rhodophyta polysaccharides, agar and carrageenan, are some of most studied and commercially applied compounds extracted from seaweeds, and, due to commercial pressure, the extraction and purification methods are already advanced in the terms of cost efficiency and economic feasibility. For example, agar and carrageenan are generally applied in the food and pharmaceutical industries as multifunctional ingredients, such as stabilizers, emulsifiers, and homogenizers, and this particularity promoted the growth in the seaweed aquaculture, mainly in Asia [18,21].

This review aims to present a comprehensive analysis and description of bioactive compounds, isolated from red seaweeds, providing relevant information about the current and potential applications of those compounds.

## 2. Fatty Acids

Algae are known to be a valuable resource of lipids [21], and phylum Rhodophyta is characterized for having a large diversity of essential fatty acids (EFAs), particularly the species that belong to the orders Gigartinales, Corallinales and Gracilariales. These groups are particularly rich in eicosapentaenoic acid (20:5ω-3) and arachidonic acid (20:4ω-6), but also in linoleic acid (18:2ω-6), a-linolenic acid (18:3ω-3) and stearidonic acid (18:4ω-3), although in a lower amount [23].

It has been demonstrated that the lipids extracted from algae, namely polyunsaturated fatty acids (PUFAs) have an essential role on the good functioning of the human body [24]. There is a high potential for the lipid extraction industry to grow. Lipids have widespread applications, such as food and dietary supplements and pharmaceutical [6,21]. However, total lipid content is present, on average, in seaweeds in concentrations between 0.60% and 4.15% [15].

In the industry, the most common extraction method is the mechanical press or hexane leaching. In order to enhance the lipids extraction yields and to be environmentally friendly, new methods have been developed, including ultrasound-assisted extraction, microwave-assisted extraction, and supercritical fluid extraction. Currently, none of the mentioned processes proved to be feasible at an industrial scale nowadays, but they are gaining new interest due to the depletion of fossil fuels, which has given rise to a demand for alternatives, of which seaweeds are a possibility. The actual techniques of fatty acid extraction have disruptive pros and cons when compared with microalgae (mainly because of the lack of fatty acids in great quantity), but they can be used to produce bioethanol and bio-oil from wet macroalgae with a viable condition [25,26].

### 2.1. PUFAs

Seaweeds are known as a low-energy, low-caloric food, and an explanation for this is the low content in lipids. Besides that, in recent years, interest in the lipid composition of marine algae has raised considerably because a big part of seaweed lipid content is constituted by ω-3 and ω-6 PUFAs, specifically α-linolenic (18:3ω-3), octadecatetraenoic (18:4ω-3), arachidonic (20:4ω-6), and eicosapentaenoic (20:5ω-3) acids [27,28,29]. Indeed, algae are essentially the only organisms able to produce long-chain PUFAs, ranging from 14 to 24 carbons, due to the presence of specific enzymes responsible for desaturation processes [29,30]. Thus, the intake of long chain EFAs, such as omega-3 and omega-6, which are abundant in most red algae [31], is mainly done by diet. PUFAs are all the fatty acids that contain more than one unsaturation (double bond) in their backbone and include the EFAs. EFAs, mainly eicosapentaenoic acid (EPA) and docosahexaenoic acid (DHA), play an important role in immunomodulation, in brain development, and in other physiological functions, such as cellular signaling, the regulation of transcription factors, and in the treatment and prevention of some cancer, ocular, cardiovascular, neurodegenerative, and autoimmune diseases [27].

The health benefiting ω-6/ω-3 ratio in macroalgae allows their use in the formulation of functional foods and nutraceuticals [29], with the influence of these fatty acids in human gene expression being known. Indeed, a dietary balanced intake of these EFAs is crucial for health and disease prevention. The omega 6/omega 3 (ω-6/ω-3) fatty acid ratio is an important parameter to evaluate the benefits from PUFAs. Previous studies reported that an omega 6/omega 3 (ω-6/ω-3) fatty acid ratio range of 3/1 to 5/1 reduces the risk of breast, prostate, colon and renal cancers. Other cases reported that the omega 6/omega 3 (ω-6/ω-3) fatty acid ratio of 2/1 to 3/1 can suppress inflammation in patients with rheumatoid arthritis. For instance, a ratio of 5/1 had a beneficial effect in patients with asthma. In addition, a fatty acid ratio of 10/1 had been reported adverse consequences [32,33].

Different methods for PUFAs’ extraction and isolation are reported in the literature, the enzyme assisted extraction, supercritical fluid extraction, and ultrasound-assisted extraction [22].

### 2.2. Sterols

Sterols are steroids whose molecule has a hydroxyl group at the C3 position and a branching side chain at the C17 position. Most of the sterols present in red algae are cholesterol and its derivatives, such as desmosterol, cholesta-4,6-dien-3-ol, and cholest-5-ene-3,7-diol [34,35,36]. These compounds are constituents of membranes and are precursors of plant and animal hormones. It has been demonstrated that they have many bioactivities, antioxidant, antiviral, anti-fungal, and anti-bacterial properties [7,8,37,38].

The sterols extraction commonly performed with a conventional solid–liquid extraction method can be conducted with a single solvent or a mixture of solvents (chloroform–methanol, hexane, methylene chloride, or acetone). Solid–liquid extraction is frequently used to extract only free sterols. The gains of supercritical carbon dioxide (CO_2_) extraction comprise its nonexistence of toxicity, chemical inertness, low cost, and ready availability. Nevertheless, this advanced extraction method is not frequently applied [7].

Phytosterols extracted from algae have widespread applications, for example, therapeutic steroids have an application on pharmaceutical production but also have nutraceutical applications through the properties of anti-cholesterol additives in foods [24].

Cholesterol is present in very low quantities in seaweeds, except in *Porphyra* sp. (Figure 1c), in which it can account for up to 8.6% of total sterols [39]. Regarding the red seaweed species, cholesterol is normally the main compound, with the highest cholesterol and total phytosterol content being reported so far for *Osmundea pinnatifida* (Figure 1d) in a study in the Portuguese coast, performed by Lopes et al. [7,40].

## 3. Terpenes

Terpenes are a non-polar compound class, classified as hydrocarbons constituted by isoprene units, and are included on the biggest class of secondary metabolites [41,42].

Terpenoids are a modified class of terpenes with different functional groups and an oxidized methyl group present or not at various positions. Thus, terpenoids are classified according to the number of carbon units. Within this variation, terpenoids hold several bioactivities with therapeutic and nutraceutical application [41].

Seaweeds comprise a wide range of halogenated biomolecules [43], and it has been reported that the genus *Laurencia* (Rhodophyta) synthetizes a high variety of sesquiterpenoid, such as 4-hydroxy-1,8-epiisotenerone, 9-hydroxy-3-epi-perforenone A, and 3-epi-perforenone [44]. These bioactive molecules have an important role in biological interactions, exhibiting antimicrobial, and cytotoxic potential [45,46,47].

*Plocamium* and *Chondrococcus* also exhibit polyhalogenated monoterpenes with therapeutic potential, likewise antimicrobial, anti-tubercular, and anti-tumor [48,49,50].

*Sphaerococcus coronopifolius* has yielded many interesting brominated cyclic diterpenes demonstrating the biological activities of these compounds. Some of them have already demonstrated antibacterial activity against Gram-positive bacteria [51] and antibacterial activity against multidrug-resistant and methicillin-resistant bacteria *Staphylococcus aureus* strains [52,53]. Still. mainly the terpernoids found in red seaweeds have demonstrated a high antitumor bioactivity [8,54,55,56]. Most of terpenoids with the variation in their structures are biologically active and are used worldwide for illnesses treatment [41].

## 4. Mycosporine-Like Amino Acid

The red seaweeds (Rhodophyta) are also producers of some small water-soluble secondary metabolites named mycosporine-like amino acids (MAAs). These water-soluble nitrogenous molecules have a high molar extinction coefficient in the ultraviolet region, establishing their photoprotective role in addition to their antioxidant and osmoprotectant action [37,57,58,59]. Given this, MAAs are often associated with skin photoprotection effectiveness. An extract of *Asparagopsis armata* (Figure 1e) containing these molecules is already incorporated in some lotions with anti-aging properties [60]. Moreover, recent studies indicated that MAAs must have other important bioactivities, such as anti-inflammatory, immunomodulatory and antioxidant properties [61].

Although these molecules are promising candidates for many possible pharmaceutical and cosmetic applications, the environmental conditions (e.g., UV radiation levels, inorganic nitrogen availability, among others) may contribute to oscillations in MMAs’ seaweed composition [57,62].

Therefore, the biochemical characterization of seaweed MAAs must be performed to ensure the bioactive properties. These molecules are colorless, neutral, water-soluble ampholytes, and they share the same chemical structure but differ in the substituents and/or presence of amino acids. The basic structure is a cyclohexanone or a cyclohexenimine chromophore [63]. Chrapusta et al. [63] stated that a better understanding of their physicochemical stability under the influence of various physicochemical stresses is needed because there is a general lack of information and the literature data indicate that MAAs do not have a common pattern.

Furthermore, due to the presence of chiral centers [61], these compounds are not easy to synthesize, which means that their commercial utilization depends on red seaweed (Rhodophyta) feedstock. There is an aquaculture method to increase the MAA content, such as increasing the irradiation with different visible light/UVR sources, and treatment with nitrate compounds. Such methods will make the seaweeds produce more photoprotective compounds [61].

An aqueous methanol extraction of lyophilized Dulse (*Palmaria palmata*) tissue, attached with a reverse-phase HPLC (high-performance liquid chromatography) allows for the separation and identification of the highly polar MAAs, such as, palythine, shinorine, asterina-330, porphyra-334, medium polarity palythinol, and the low polarity usujirene in the present study [64].

Previous studies reported MAAs’ presence in red seaweeds (Rhodophyta), namely *Chondrus crispus* (Figure 1f), *Palmaria palmata*, *Gelidium* spp., *Pyropia* spp. (formerly known as *Porphyra* spp.), *Crassiphycus corneus* (formerly known as *Gracilaria cornea*), *Asparagopsis armata*, *Solieria chordalis*, *Grateloupia lanceola*, and *Curdiea racovitzae* [64,65,66].

## 5. Proteins

Seaweeds synthesize a variety of proteins, in which its composition, structure, and bioactive potential evaluation is not much very exploited and elucidated [67]. Red algae present a high content of protein, around 35% to 47% [6,68], which is higher in comparison with terrestrial plants resources, for example, soybean has a protein content with average values between 30% and 40% dry weight (dw) [69].

The ratio between essential amino acids and non-essential amino acids (EAAs/NEAAs) evaluates the allocation of essential and non-essential amino acids on macroalgae proteins. Rhodophyta species present many essential amino acids [70].

In red algae the EAA/NEAA ratio is 0.98–10.2 (EAA/NEAA) [71]. Moreover, brown algae (Phaeophyceae), for example, *Ascophyllum nodosum*, present a lower EEA/NEAA ratio of 1–1.06 [70].

Thus, the inclusion of edible seaweeds on a daily diet is considered to be a nutraceutical food since the protein values are similar or superior to legumes and soybean, 30–40% dw [72].

Proteins and amino acids synthesized by red seaweeds also have great pharmaceutical potential as they have been reported to have multiple bioactivities [70,73,74,75,76,77,78,79]. Illustratively, pepsin, which digests from *Pyropia yezoensis* (formerly known as *Porphyra yezoensis*), has been reported to have an angiotensin-converting enzyme (ACE) inhibitory effect, anti-mutagenic, blood sugar reducing, calcium precipitation inhibition, decrease of cholesterol levels, antioxidant, and improved hepatic function activities [67].

The improvement of seaweed protein extraction methods could enhance the protein content [80].

Protein extraction methods comprise conventional as well as novel extraction technologies, such as enzyme-assisted and microwave-assisted extraction [81]. The content and biodisponibility of protein is dependent on the extraction method, for example, the hydrolysis with a blend of cellulase and xylanase improved the yield of protein extraction in *Palmaria palmata* [80]. Other studies revealed that the protein extraction using *P. palmata*, using osmotic shock, high share force, and alkaline and polysaccharide treatments has presented a better protein recovery [67]. The digestibility of seaweed proteins is inhibited due to their entrapment in the cellular matrix [42].

The literature reports the presence of amino acids such as glutamic acid, taurine, threonine, arginine, alanine, and aspartic acid in the seaweeds *Chondrus crispus*, *Gracilaria* sp., *Osmundea pinnatifida*, and *Porphyra* sp. [70]. In this context, Vieira et al. [70] recorded particularly higher values of the EAA/NEAA ratio on the Portuguese coast of *Gracilaria* sp., presenting values between 1.47 and 1.74 (EAA/NEAA ratio) and *C. crispus* between 1.58 and 1.10 (EAA/NEAA ratio).

Some unusual amino acids present on seaweeds, such as laminine and kainoids, have been investigated for their potential to treat neurophysiological disorders, such as epilepsy, Alzheimer’s, and Parkinson’s diseases [75].

In general, essential amino acids represent 25–50% of the total amino acids in seaweed dry weight [68].

## 6. Pigments

Seaweeds pigments’ composition is distinctive from specific algal taxa. Red seaweeds are characterized by the presence of phycobiliproteins and carotenoids [82].

Pigments are polar compounds associated with phycobilisomes, which are a regulatory protein complex, with the function of light harvesting and have a pivotal role on photosynthesis by performing light harvesting [20,83,84,85].

Phycobilins are a designation of open chain tetrapyrroles (four pyrrole derivative compound) present as photosynthetic accessory pigment that can absorb light from visible radiation, which is poorly absorbed by the chlorophyll, thus permitting the seaweeds living in deep-water [86].

The chromophore is the part of a molecule responsible for the color (not absorbed by the chromophore) and giving the color to the molecules specifically [87].

Carotenoids are a family of natural pigments extensively present in the seaweeds, mainly in brown seaweeds, of which the principal function is their contribution to the light-harvesting process, the filtering of deleterious light radiations and the antioxidant activity. Chemically, the carotenoids can be classified between carotenes (pure hydrocarbons) or xanthophylls (oxygenated carotenes). Carotenes and xanthophylls are classic tetraterpenoids. Carotenoids are biosynthesized in plastids that present their own organelle membrane and behave in a lipophilic environment with other biomolecules [88].

Additionally, pigments act as photoprotective agents, due to their role in the seaweed antioxidant defensive mechanism preventing the reactive oxygen species (ROS) effect induced by the UV radiation and other abiotic factors [82,83,89].

A study conducted by Zepeda et al. [90] verified that the light quality will cause modifications on the seaweed growth rate and pigment synthesis. By using different LED light treatments, it was possible to correlate the antioxidant activity with pigment concentration. More light intensity leads to a higher pigment biosynthesis. This type of defense response is related to the species’ ecology, as a survival mechanism under the environmental conditions of its wild habitat [90]. For instance, in the wild it is expected that tropical seaweeds have a higher content of pigments due to the higher incidence of UV light underlying the direct path of solar radiation throughout the atmosphere [83]. Seaweed farming could support the optimization of seaweed pigment yield through the regulation of light type and intensity, in order to have a lower or higher yield of pigments. This will allow a wide range of future applications of red seaweed compounds or extracts for various industries [90].

Currently, the extraction and purification methodologies are evolving in order to be more efficient and cost-effective because the pigment separation techniques are especially expensive.

### 6.1. Phycobiliproteins

Phycobiliproteins (PBPs) are a family of light-harvesting pigment–protein complexes. These compounds can efficiently transmit light energy to chlorophyll *a*, and thus algae are capable of undergoing photosynthesis [91,92]. From their light absorption properties and types of bilins, Phycobiliproteins are divided into four main groups: phycoerythrins (λ_max_ 540–570 nm), phycocyanins (λ_max_ 610–620 nm), phycoerythrocyanins (λ_max_ 560–600 nm), and allophycocyanins (λ_max_ 650–655 nm) [93]. Phycobiliproteins from red seaweeds form complexes between proteins and covalently bound phycobilins, which act as chromophores (the light-capturing part). Phycobiliproteins are the most important compounds in the phycobilisomes of red algae [94,95].

Cyanobacteria and seaweeds that belong to phylum Rhodophyta are the main resources to extract phycobilins. This compound has pharmaceutical potential, due to its antioxidant activity [21,96]. It also has potential in the food industry as a nutraceutical compound and on cosmetic industry as a colorant [96].

In general, high quantities of phycobiliproteins could be extracted fast from seaweed through the water extraction method [97,98].

#### 6.1.1. Phycoerythrin

Phycoerythrin is a major associate pigment in the phylum Rhodophyta, so the red color is due to its high concentration in the seaweed. The difference of the several absorption wavelengths of phycoerythrin can be used for the spectroscopy identification of which type of phycoerythrin is present in the seaweed, which is a quick detection method of the pigment [63,64,65]. However, R-phycoerythrin (R-PE) extraction needs to have a purifying process, which is expensive and laborious [91].

Usually, red seaweeds are able to grow in deep seawater largely due to their high quantity of phycoerythrins, which have efficient absorption of light wavelength from 450 to 570 nm [99]. Phycoerythrins are divided into three main groups: B-phycoerythrin (λ_max_ = 565 nm, 546 nm, and a shoulder at 499 nm), C-phycoerythrin (λ_max_ = 565 nm), and R-phycoerythrin (λ_max_ = 565 nm, 498 nm, and a shoulder/peak at 540 nm). R-phycoerythrin is the most abundant phycobiliprotein found in red algae [100]. *Gracilaria gracilis* can be proposed as a novel industrial source of phycobiliproteins, namely phycoerythrin, since the concentration produced could vary between 3.6 mg/g and 7 mg/g dw according to the season and the *C. crispus* have a content of 528 mg/kg dw [3,101]. *Gelidium amansii* collected in South Korea have recorded 53 µg/g dw of phycoerythrin [102].

The sales price of R-PE ranges from about €50 mg/L to about €146 mg/L depending on the purity level of the molecule [91].

The process of phycoerythrin extraction and purification can be expensive and time consuming, so the current challenge is to reduce costs and time. Nguyen et al. [91] applied with success the ion-exchange chromatography to purify the phycoerythrin derived from *G. gracilis* and *Grateloupia turuturu*, reducing time and costs. This technique is cost efficient and economically viable because phycoerythrin is extracted with a nitrogen and phosphate buffer and purified in an ion-exchange chromatography based on the work of Munier et al. [103].

Phycoerythrin has recently entered as an ingredient in food, medical, and cosmetic industries as a natural colorant and is used as fluorescent markers in biomedical research [82,91].

#### 6.1.2. Phycocyanin

A blue-colored light-harvesting pigment found in seaweeds is phycocyanin. Phycocyanin is a conjugate polypeptide chain [87,92,104] with a chromophore phycobilin.

In the last 20 years, research performed with microalgae to phycocyanins large-scale purification processes achieved significant improvements, however, this process is unstable, limiting its industrial feasibility [103,105].

The phycocyanins extraction techniques include the cell disruption by mechanical (e.g., ultra-sonification and bead mill) or non-mechanical methods (chemical osmosis and a repeated freeze–thaw cycle). The non-mechanical procedure is used in laboratory scale but is not feasible applied to the industry, and further research needs to be done in order to scale-up these techniques [92].

The main task to be done relatively to the phycocyanin extract, is the purification methods that are the main target of studies about phycocyanin. Chromatographic methods are the most used because they can be applied to industrial production and they are currently the most effective methods. The ion-exchange chromatography is supported in the electrostatic interaction force between the charged solute and the ion-exchange solvent. Due to the negative charge of phycocyanin in a weakly acidic medium, it is extensively purified by anion-exchange chromatography [92].

Francavilla et al. [3] demonstrated that the less abundant phycobiliprotein in *G. gracilis* is phycocyanin, displaying a concentration that fluctuated from 3 mg/g dry weight in January to 0.7 mg/g dry weight in October. Pina et al. [101] analyzed the *C. crispus* collected in Spain with a present value of 149 mg/kg dw. Sukwong et al. [102] extracted 56 µg/g dw of phycocyanin from *G. amansii*.

Studies have demonstrated the effectiveness of these molecules at improving the immune system in the human body and promoting the regeneration of animal blood cells [92]. In fact, they are being widely used in the fields of molecular biology, immunology, cytology, and molecular diagnoses [79,96,106].

Several studies are being carried out on the bioactivities of phycocyanin, including in vitro anticancer activity, chemotherapy sensitiveness, photosensitized tumor suppressor activity, anti-inflammatory effect, anti-oxidative, and anti-irradiative effects. The immunomodulatory function of phycocyanin promotes cell growth and has a neuroprotective effect [92,107,108]. All these properties make phycocyanin an interesting biomolecule and an effective ingredient for novel functional food products, and also for cosmetics and pharmaceuticals [92,96].

#### 6.1.3. Allophycocyanin

Allophycocyanin is a phycobiliprotein located at the core of the phycobilisome. The allophycocyanin is isolated and purified and is used in the spectrophotometric analysis due to the trimer of allophycocyanin having an unusual absorption maximum at 650 nm with a shoulder at 620 nm, while the monomer has an absorption maximum at 615 nm [109].

Allophycocyanin extraction has low yields because allophycocyanin is a minor constituent of phycobiliproteins [110,111]; therefore, it is challenging to achieve a high purity level due to presence of other proteins that are co-extractables.

The mentioned challenge could be overcome with the development of efficient methods of extraction and purification. Thus, the chemical/enzymatic cell disruption extraction method proved to be the best method to extract allophycocyanin with a high purity [112].

Francavilla et al. [3] extracted between 3.5 mg/g dry weight and 1.5 mg/g dry weight of allophycocyanin from *G. gracilis* (samples from January and October, respectively). Dumay and Morançais [113] report that the allophycocyanin can vary between 0.5 and 5.28 mg/g dw.

This phycobiliprotein have reported to exhibit antioxidative [114], anti-inflammatory [96], antitumor [115], anti-enterovirus [116], and hepatoprotective [117] properties.

### 6.2. Carotenoids

Carotenoids are other pigments produced by algae. In red seaweed, the main carotenoids present are α and β-carotenoids.

In less quantity, other carotenoids can be found, such as antheraxanthin, cryptoxanthin, lutein, violaxanthin, and zeaxanthin [82,84].

When compared to other secondary pigments present in the red seaweeds, carotenoids are the most extensively investigated. Several methodologies have been developed for carotenoid extraction, such as the supercritical fluid extraction [118]. These methods are under investigation nowadays to be more cost-effective and to obtain a quality product [118].

Like other pigments present in red seaweed, carotenoids are also biologically active compounds, possessing an antioxidative [88,119], anti-inflammatory [119,120], and antitumor properties [121], and diminish the risk of ophthalmological diseases in humans [122,123]. Recently, carotenoids have gained interest due to their antioxidant action. In this context, carotenoids could be used to reduce the incidence of some chronic diseases. Moreover, carotenoids are thought to protect cells from oxidative stress by quenching single oxygen damage with various mechanisms [38,124].

Red algae do not own an exclusive carotenoid profile. Modifications of carotenoid composition are related to the presence or absence of specific minor carotenoids but mainly in the xanthophyll that represents the major carotenoid. Schubert et al. [125] did a wide screening of red algae carotenoids that demonstrated that Gigartinaceae have more content of lutein, the same result as the Gelidiales. The Gracilariales have more content of zeaxanthin and Corallinales the main carotenoid is antheraxanthin.

Currently, algal carotenoids are already incorporated in daily products, such as food coloring, feed additives (e.g., aquaculture), components of cosmetics, and pharmaceuticals [1,24].

## 7. Phenolic Compounds

Phenolic compounds are chemically characterized as molecules containing hydroxylated aromatic rings, having the hydroxyl group attached directly to the phenyl, substituted phenyl, or another aryl group [126]. The ecological function of phenolic compounds in red algae has been barely investigated; however, in other organisms they are known as antioxidants, hormones, cofactors, or defense compounds. In general, phenolic compounds are detected at low concentrations in the genus *Gracilaria*. Among the few phenolics already identified, there are bromophenols and benzoic acids [127]. Bromophenols are phenolic compounds found in red algae, with bromine substituent indistinct degrees [128]. The bromophenols already identified in the genus *Gracilaria* are simple bromophenols with just one benzene ring, such as 2-bromophenol, 4-bromophenol, 2,4-dibromophenol, 2,6-di-bromophenol, and 2,4,6-tribromophenol [129]. The biosynthesis of these compounds has not been completely understood yet, but tyrosine may be the precursor [129].

They act as intermediates in the biosynthesis of many secondary metabolites, being also important precursors for the industrial synthesis of many other organic substances. Inclusively, the salts of phenolic acids are used as industrial food preservers. Some of these compounds have already been identified in the genus *Gracilaria*, such as benzoic acid, p-hydroxybenzoic acid, salicylic acid, gentisic acid, protocatechuic acid, vanillic acid, gallic acid, and syringic acid [34,130,131]. Most phenolic compounds possess a broad variety of biological activities, as the bromophenols have anti- diabetic, antioxidant, and anticancer properties [128].

Farideh Namvar and colleagues [132] investigated the effect of *Kappaphycus alvarezii* (formerly known as *Eucheuma cottonii*)-polyphenol-rich extract (ECME). The concentrations used in this study did not show a toxic effect on the normal cells; however, it was cytotoxic to the MCF-7 cancer cell line. This suggests that the ECME’s active substance may affect cancer-associated receptors, cancer cell signaling molecules, or the gene expression of the cancer cells that triggers mechanisms causing cancer cell death [132].

## 8. Polysaccharides

Polysaccharides are the main constituent element of seaweed cell wall, representing 40–50% of its dry matter [68]. However, the environmental and ecological conditions play a pivotal role on the biosynthesis of these compounds. For instance, when seaweeds grow in an environment characterized by calm waters, there is a lower production of polysaccharides. The main function is to protect from dryness and physical aggression from biotic and abiotic factors, such as rough waters and waves [133]. The polysaccharides flow as a structural carbohydrate in the cell wall and in the intercellular spaces [76].

The structure and rheological of the polysaccharides are dependent on species, life-stage, growth, environment, and the extraction method [134].

Due to polysaccharides quantity on seaweed weight and its widespread applications, the market value of polysaccharides-based products is high based on their technological features [68].

The polysaccharides in the red seaweed are mainly sulphated galactans. Much like the agar, carrageenan, and porphyran, this type of polysaccharide presents a main structure base of galactose units with a variation at the level of sulphation of the galactose units [135]. This sulphation level can modify the bioactivity of the polysaccharide, such as antioxidant, anti-tumoral, or the treatment of hypertension [135,136].

### 8.1. Agar

Agar is a phycocolloid composed mainly of agarose and agaropectin units. Agar is a sulphated polysaccharide, composed of α (1-4)-3, 6-anhydro-L-galactose and β 9(1-3)-D-galactose residues [137].

Agar is mainly extracted from *Gelidium* sp. and *Gracilaria* sp. [137,138]. Moreover, it is the most used phycocolloid compound derived from red seaweeds. The utilization of this phycocolloid is mainly in commercial and scientific (biotechnological) areas, such as adhesives, suppositories, capsules, textile printing/dyeing, and bacteriology assays [77,135].

According to the molecular composition and purity degree, the agar quality can be distinct. Additionally, the agar quality and its content depend on the species of red algae; species that belong to the genus *Gelidium*, *Gracilaria*, and *Pterocladiella* are considered the most productive seaweeds. Additionally, the physiochemical property is closely related to environmental parameters, the growth and reproductive cycle of the seaweed used as its source. Although agar is used at a commercial level outside the hydrocolloid industry, it is recently used in medicinal and pharmaceutical applications against cancer cells, since it can promote the apoptosis of these cells in vitro [139].

Previous studies demonstrated that agar obtained from cold-water extraction of *Gracilaria* species shows anti-tumoral activity. Moreover, hydrolysates of agar (agaro-oligosaccharides) have been demonstrated to have activity against glycosidase and antioxidant ability [140].

Besides, agar extracted from *Gelidium* is better quality and is easily extracted with boiling water. The gelling ability of agars from *Gracilaria* sp. can also be enhanced by an alkali pre-treatment to convert α-L-galactose-6-sulfate into 3,6-anhydro-α-L-galactose. This process reduces the sulfate content, improving the gelling properties as evidenced by higher gel strength, gelling and melting temperatures, and viscosity [141].

The high-quality agar (agarose) is used in molecular biology for separation techniques like electrophoresis, immunodiffusion, and gel chromatography. It is known for the manufacturing of capsules for industrial application and it is also used as a medium for cell culture [135].

The medium quality agar is used in biotechnological and research areas, like in biological culture media as the gel substrate, whereas the low-quality agar is used in food products like candies, fruit juice, frozen foods, bakery icing, or meringues. Agar industrial application also includes paper coating, adhesives, textile printing dyeing, impressions, or casting [77].

The commercial form of agar approved for food industry by the Food and Drugs Administration (FDA) and the European Food Safety Agency (EFSA), in Europe is coded E-406 by the Commission Regulation No 257/2010 [142,143].

They are also important and are used in the field of medicine and pharmaceuticals as a bulking agent, an anticoagulant agent, laxatives, capsules, and tablets [143].

*G. gracilis* is the third largest species of the phylum Rhodophyta that is used mainly for the production of agar with the first two species belonging to *Gelidium* genus [18,144]. So, the genera more used by the agar extraction industry are *Gelidium*, *Gracilaria*, and *Pterocladiella* [145].

### 8.2. Carrageenan

Carrageenan is a phycocolloid with a defining characteristic of alternated galactose and 3,6-anhydrogalactose sugars linked by alternate α-1,3 and β-1,4 glycosidic linkages [146,147]. There are different types of carrageenan, which are present in different species of Gigartinales (Rhodophyta). Carrageenans can be classified as λ (lambda), κ (kappa), and ι (iota) according to the number of sulphated groups of the galactose unit, where number, chemical location, and arrangement of these groups defines carrageenan function and property [148].

Intrinsic carrageenans hold a complex hybrid chemical constitution that differs according to the environmental and ecological conditions to which the seaweed is exposed. Generally, carrageenans are composed by a combination of galactans that could possess several types of carrabiose [149,150]. Thus, seaweeds do not produce these flawless and clean carrageenans, but, instead, they produce a full variety of hybrid configurations of carrageenan that are also dependent on the species life cycle [151].

The carrageenan extraction technique will primarily control the purity of the carrageenan extract. When carrageenans are manufactured by the alcohol extraction technique, they hold nearly 90% of anhydrous carrageenan, 8% moisture, and 2% inorganic salts (mainly chlorides), whereas those extracted by the gel press technique contain about 77% anhydrous carrageenan, 8% moisture, and up to 15% inorganic salts [152].

This hydrocolloid has a molecular weight variable within 30 and 5000 kDa but the median molecular weight of carrageenans is between 200 and 800 kDa [146,152]. This hydrocolloid does not have nutritional value, because the human body does not possess enzymes to metabolize carrageenan and it is not digested by the human digestive tract, meaning that it is regarded as dietary fiber [146].

The commercial forms of λ-, κ- and ι-carrageenans were approved for the food industry by the Food and Drugs Administration (FDA) and the European Food Safety Agency (EFSA) [146,152]. Other types of carrageenans are not approved for the food industry but are used in biomedical and pharmaceutical studies and the R&D of new products, therapeutics, and technologies [146,152].

In the food industry, the carrageenan application is regulated by the Commission Regulation (EU) No 231/2012 in Europe, which states that commercial carrageenan (E 407) fundamentally involves potassium, sodium, magnesium, and calcium sulphate esters of galactose and 3,6-anhydrogalactose polysaccharides [152]. In the opposite direction, the poligeenan, a degraded ι-carrageenan with typical molecular weight of 10–20 kDa, has not been authorized in food applications inside of the European Union area, because the poligeenan is also known as an artificial product derived from carrageenan that is associated with adverse effects [146,153]. So, more studies and the development of methodologies are needed to give security to this type of degraded carrageenan.

Carrageenans are water-soluble polymers, their solubility being determined by the type of carrageenan, temperature, pH, and the counter ion in the dissolving solution, κ-carrageenan being the most soluble. The sodium salt of κ-carrageenan is soluble in cold water, but the potassium salt is soluble only by heating. ι-carrageenan has an intermediate solubility [154]. All types of carrageenan are insoluble in organic solvents including alcohols and ketones [155].

Carrageenans are one of the main natural texturizing agents used in food applications (dairy products, jellies, pet foods, sauces) that are considered safe food additives [148,154]. When used in food products, carrageenan has the EU additive E numbers E407 or E407a when present as “processed eucheuma seaweed” [152].

They are also used in pharmacological formulations, cosmetics, as biomedical polymer compounds, or as lubricant [148,154,156]. Overall, carrageenans principal functions in industry are as a gelling, stabilizing, and viscosity-building agent.

In the last decades, the biological potential of carrageenans has been explored. It has been proven that carrageenans have anticoagulant and antithrombotic properties, with λ-carrageenan showing higher anticoagulant potential than κ-carrageenan [157]. Carrageenans have also been shown to selectively inhibit many enveloped virus [158], having antioxidant properties [159] and anti-tumor potential [160,161].

The carrageenans are extracted from different species of the Gigartinales (Rhodophyta), depending on the type of carrageenan necessary. Kappa (κ)-carrageenan is extracted from *Kappaphycus alvarezii* (commercial identified as “cottonii”), while iota (ι)-carrageenan is generally extracted from *Eucheuma denticulatum* (commercial identified as “spinosum”). The other commercial carrageenan, the lambda (λ)-carrageenan is extracted from species belonging to the *Gigartina* and *Chondrus* genera (commercial identified as “Irish moss”) [161].

### 8.3. Porphyran

*Porphyra* sp. (Rhodophyta) is characterized by the presence of porphyran, an anionic polysaccharide. The mentioned compound is a galactose, highly substituted by the 6-O-sulfation of L-galactose units and the 6-O-methylation of D-galactose units.

Historically, countries of East and Southeast Asia are the biggest consumers of seaweeds, due to the culture of eating sushi. In this market, seaweeds belonging to *Pyropia*/*Porphyra* genus are known by “Nori” and it is, usually, commercialized as a dry product [73,162,163].

There are different techniques to this compound extraction, such as hot water extraction, radical degradation, and ultrasonication [164].

Porphyrans have been reported to have the following properties: hypolipidemic, anti-cancer, and anti-inflammatory in humans. The anti-inflammatory properties could be confirmed by the inhibition of nitric oxide (NO) production in macrophages and by blocking NF-B activation in the mouse macrophages of RAW264.7 cells that were stimulated with porphyrans [165].

A previous study demonstrated porphyrans’ antioxidant activity in mice by preventing hyperlipidemia [166].

Additionally, another group of research presented a study in which lipid synthesis in HepG2 cells was repressed, which reduced the secretion of apolipoprotein B100 by the action of porphyrans, demonstrating its hypolipidemic effect [167].

Research from Kwon and Nam shows that porphyrin is not toxic for cells, otherwise it is toxic for cancer cells being an inductor of cell death depending on the dose [168].

## 9. Vitamins

Vitamins are micronutrients that play an essential role in metabolic paths as an enzyme co-factor precursor. In this context, there are some essential vitamins that the human body is not able to produce, being mandatory to get those vitamins through food sources.

Most of terrestrial plants are unable to synthetize vitamin B_12_.Thus, seaweeds such as *Porphyra umbilicalis* and *Crassiphycus changii* (formerly known as *Gracilaria changii*) (Rhodophyta) have high quantities of vitamins, especially vitamin C, and also contain all the essential and non-essential vitamins. Red seaweeds are also an important resource rich in vitamin A (carotenoids) [69].

The interaction between prokaryotes, which synthesize vitamin B_12_ and the seaweed surface, promotes this vitamin level present in the macroalgae. Vitamin B_12_ content is reported to be higher in microalgae such as *Chlorella* (Chlorophyta) *and Spirulina* (Cyanobacteria), presenting 33.3 and 15.3 g/kg of fresh weight, respectively. In contrast, *Porphyra* sp. presents 1 g/kg fresh weight of vitamin B_12_. Castillejo et al. [169] tested seven seaweed and two microalgae, despite the low levels of vitamin B_12_ in *Porphyra* when compared to microalgae. This quantity would be enough to suppress the vitamin B_12_ daily need of a person when added to a smoothie. The active vitamin B_12_ coenzymes comprised about 60% of total vitamin B_12_ in Nori and *Chlorella* (Chlorophyta) supplements. This evidence shows the utilization of seaweed as a vitamin B_12_ supply.

It is important to take into consideration that the process of drying or the lyophilization can change the seaweed vitamin bioavailability. For example, air-dried *Pyropia tenera* has produced biologically inactive B_12_. In opposition, the lyophilization process might have improved nutritional quality due to the presence of active vitamin B_12_ [169].

Kendel et al. [170] demonstrated that *Grateloupia turuturu* (harvested in French Atlantic Coast) has α-tocopherol (vitamin E) and phytonadione (vitamin K_1_), proving the high nutraceutical power.

## 10. Minerals

The fact that macroalgae are so rich in diverse minerals essential for human health makes seaweed an important resource to be applied as nutraceuticals. Iron (Fe) and Iodine (I) are the main mineral compounds prevalent in seaweeds. Commonly, seaweeds hold a similar mineral concentration of seawater.

However, seaweed composition in minerals is variable in response to biotic and abiotic factors.

Minerals perform a structural role and are linked to several metabolic pathways as cofactors of different catalytic metalloenzymes [14].

A correct consumption of minerals is important to maintain a good diet nutritionally. Thus, it will help to avoid illnesses related to nutritional deficiency, cardiovascular problems, degenerative diseases, and cancer [171,172].

The inclusion of iodine in diet is fundamental to the synthesis of thyroid hormones—thyroxine and triiodothyronine—that have a key role on the regulation of physiological processes of human beings [173].

Additionally, just like the iodine, the iron plays a key role on the cellular functions, significantly in the oxygen transportation and as a constitutional part of several enzymes involved in DNA synthesis and electron transport [174,175].

Zinc (Zn) is an essential mineral that has a structural role in metalloenzymes and takes part in DNA and RNA synthesis, gene expression, and cell division [14,176].

Manganese (Mn) is also necessary to the good function of several metabolic processes that request large energy quantities, such as protein, lipid, and carbohydrate metabolism. Thus, this element occurs at brain, retina, liver, pancreas or kidneys. Moreover, it is needed for the pathways that help blood clotting and hemostasis [177].

Minerals are fundamental for the good performance of the human body, and *Palmaria palmata* (Rhodophyta) could be a good iron resource because it contains 800 mg/kg dw. In the European Union, the recommended iron uptake dosage is 14 mg/day [14,15].

Iodine content in *P. palmata* (collected in the North Atlantic area) can be near 293 µg/g dw, and in the *Porphyra* sp. (collected in Japan) it was only 16 µg/g dw. The worldwide dosage intake recommendation for iodine is the 0.15 mg/day [14,178,179].

Zinc is present in a reasonable quantity in red seaweeds, such as *C. crispus* (Denmark) 74 mg/kg dw, *Porphyra* spp. (France) 82 mg/kg dw, and *Gracilaria* spp. collected in Greece, which have 95 mg/kg dw of zinc content, the recommended dosage being 10 mg/day [14,180].

The higher content of the trace mineral manganese is reported to occur in red seaweeds, especially in *C. crispus* (Denmark), *P. palmata* (Spain), and *Gracilaria* spp. (Denmark) that have 653, 233, and 502 mg/kg dw, respectively, where the dosage recommended in Europe is 2 mg/day [14,124,180].

These values will be different due to the differences in the concentrations of minerals in seawater.

## 11. Conclusions and Futures Perspectives

Seaweeds are turning into one of the most attractive natural sources for the attaining of compounds, having a high potential for the development of novel therapeutic and nutraceutical products. Seaweed present advantages over other sources of bioactive compounds due to their cost, feasibility, and environmentally friendly production processes, as well their high extraction yield and, most importantly, the availability of biomass in natural ecosystems. Some of the compounds synthetized by seaweeds are extensively used in food applications for many properties to enhance food quality. For instance, seaweeds are applied as a functional ingredient in commercial usage as a stabilizer, emulsifier, thickening agent, texture modifier, phytochemicals (enriched with vitamins), and as dietary fiber.

Currently, one of the main goals is to guarantee that wild seaweed species and seaweeds produced in aquaculture have a similar or higher concentration of biomolecules in order to ensure feedstock for extraction and ecological sustainability. There are some species from phylum Rhodophyta whose feasibility potential is higher, such as *Gracilaria gracilis*, *Kappaphycus alvarezii* and *Pyropia*/*Porphyra* spp.

Seaweed aquaculture will be highlighted in the next years to answer this growing market and its high demand.

The road to the future should pass by the biomolecules identification and an attempt to replicate in an aquaculture system. For instance, it is what happens with seaweed used for agar and carrageenan extraction or more simply for direct unmanufactured food.

The extraction methods need to be optimized to be more ecofriendly and cost-effective, with a high purity level to support the industries that want to work with these biomolecules and to obtain more than one compound from seaweed biomass to create add-value products.

This review was a compendium that will support the comprehension of what are the main compounds extracted from red seaweeds, its extraction methods, and the most targeted species under study. Further research indicates that future investigation will be focused on improving seaweed farming methodologies and the optimization of biomolecules extraction methods.
